# A New Lab Developed Real Time PCR Assay for Direct Detection of C. Difficle from Stool Sample without DNA Extraction

**Published:** 2016-09

**Authors:** Brandon Li

**Affiliations:** Sino US Gene, Inc. 1452 #C, West Holt Ave. Pomona, CA 91768, USA

**Keywords:** Clostridium difficile, real-time PCR, enzyme immunoassays, tcdB

## Abstract

*Clostridium difficile *is a major cause of nosocomial antibiotic-associated infectious diarrhea and pseudomembranous colitis. Detection of *C. difficile* by anaerobic bacterial culture and/or cytotoxicity assays has been largely replaced by rapid enzyme immunoassays (EIA). However, due to the lack of sensitivity of stool EIA, we developed a multiplex real-time PCR assay targeting the *C. difficile* toxin genes *tcdB*. stool samples from hospitalized pediatric patients suspected of having *C. difficile*-associated disease were prospectively collected. Three testing modalities were evaluated, including enriched culture, cepheid Xpert and real-time Pcr (*tcdB*) on stool samples performed with *tcdB* gene-specific primers and hydrolysis probes. A total of 150 de-identified clinical specimen were analyzed. The sensitivities of stool real-time Pcr were 95% against cepheid Xpert C. difficile and 93% against enriched culture respectively, with a specificity of 97% and 94%. The lower limit of detection of the stool real-time PCR was 0.5 cFU/ml of per reaction for *tcdB*. Direct detection of *C. difficile* toxin genes in stool samples by real-time Pcr showed performance comparable to enriched culture. Real-time PCR of DNA from stool samples is a rapid and cost-effective diagnostic modality for patients that should facilitate appropriate patient management.

## INTRODUCTION

*Clostridium difficile*, a Gram-positive spore-forming bacillus, is the most common identifiable etiologic agent of antibiotic-associated diarrhea (13, 18). Initially described as a member of the commensal microbiota of neonates, *C. difficile* was identified as a causal agent of antibiotic-associated diarrhea in the 1970s (3, 11). The clinical presentation of *C. difficile*-associated disease (CDAD) can range from asymptomatic carriage in the gastrointestinal tract, mild diarrhea, and potentially fatal pseudomembranous colitis (13, 18). Symptoms occur secondary to the production of two exotoxins, toxin A and toxin B, which disrupt the integrity of the colonic mucosa (24).

Alarming changes in the epidemiology of CDAD, including an increase in both the incidence and severity of the disease, have highlighted concerns about patterns of *C. difficile* infection (17, 18, 19, 21). Analysis of U.S. hospital discharge data revealed that the national rates of CDAD doubled from 2000 to 2003 (17). In 2004, the Centers for Disease Control and Prevention reported that the mortality rate related to CDAD increased from 5.7 deaths per million individuals in 1999 to 23.7 deaths per million individuals (21). In addition to the profound morbidity and mortality, CDAD is also generating a substantial economic burden, with estimates ranging from $1.3 million to more than $3 billion annually (8, 16, 20). Due to the formidable impact of CDAD on the U.S. health care system, rapid and accurate diagnosis is essential for the timely enactment of infection control and treatment measures.

The changing epidemiology of *C. difficile* infections in the pediatric population is a serious concern. While benign neonatal colonization with toxigenic *C. difficile* is a well-documented phenomenon, recent studies have suggested an increased incidence of CDAD in children (2, 14, 22, 25). A large study encompassing data collected from 22 children’s hospitals in the United States reported an increased prevalence of CDAD in children, including infants (increased by 53% from 2001 to 2006, with 26% of patients with CDAD ≤1 year of age) (14). Utilizing CDAD data from the Agency for Healthcare and Research Quality, a similar study noted that the highest number of CDAD hospitalizations occurred in patients ≤1 year of age (25).

Initial strategies to detect *C. difficile* consisted of anaerobic stool sample culture, usually with cycloserine-cefoxitin-fructose agar (CCFA) or a similar medium with or without a pretreatment alcohol shock step (7). Although this modality was quite sensitive and specific for detecting *C. difficile*, it took up to 5 days to confirm a negative culture and it did not discriminate between toxigenic and nontoxigenic isolates without further testing strategies. Furthermore, colonies with indeterminate colony characteristics were tested with L-proline-aminopeptidase (PRO Disc) or other biochemical tests to ensure the accurate identification of *C. difficile* (9, 10). The development of the cell culture cytotoxicity assay circumvented stool sample culture by observing cytopathic effects of toxin B directly on cultured cells (4, 6). The cell culture cytotoxicity assay requires a neutralization step for specificity and maintenance of toxin-susceptible mammalian cell lines, and it takes 48 to 72 h to perform the assay (1, 5). Rapid antigen detection assays, consisting of common antigen testing (glutamate dehydrogenase) and toxin immunoassays, have largely replaced culture and the cytotoxic assay; however, neither type has the desired sensitivity or specificity to reliably confirm or rule out CDAD without the need for either serial testing or subsequent testing modalities. Therefore, real-time PCR is being investigated as the preferred diagnostic modality due to its rapid turnaround time and track record of superior sensitivity and specificity.

Toxigenic strains of *C. difficile* contain a 19.6-kb pathogenicity locus (PaLoc) that includes five contiguous chromosomal genes responsible for the development of CDAD—*tcdABCDE* (24). *tcdA* and *tcdB *encode exotoxins A (enterotoxin) and B (cytotoxin), respectively; *tcdC* and *tcdD*encode negative and positive regulators, respectively, that control the level of toxin production; and *tcdE* is purported to encode a holin-like protein thought to facilitate toxin release from the bacterial cell wall (24). Because toxins A and/or B are implicated in CDAD and genetic diversity of the PaLoc has been reported (23), we developed and clinically validated one hydrolysis probe real-time PCR assays targeting the *tcdB* genes (12, 15, 24). While the molecular methods utilized by this assay were not novel, the application of molecular testing for *C. difficile* infection is unique when the stool sample could be tested directly without nuclear acid extraction. This will greatly facilitate quick testing of c. difficile in clinical setting.

## MATERIALS AND METHOD

C. difficile Strains: The following strains were used for LoD study: C. difficile ATCC 43255 (ZeptoMetrix), C. difficile NAP1A (ZeptoMetrix).

Extraction, Real-time PCR Amplification and Detection: Lab developed C. difficile Direct Kit contains all reagents for on-board extraction and real-time PCR amplification.

Fifty μL of C. difficile Direct reaction mix was loaded into the reaction port and 50 μL of sample was directly loaded into the sample port on the Amplification cell. All testing was performed using real time PCR. Assay time is about 60 minutes.

Limit of Detection (LoD): The LoD for each C. difficile stock was determined as the lowest concentration with ≥95% detection in negative stool matrix.

Reproducibility: Thirty-six replicates of the following contrived panel in negative stool matrix were tested: C. difficile Low Positive (ATCC 43255), C. difficile Medium Positive (ATCC 43255), C. difficile Low Positive (NAP1A), C. difficile Medium Positive (NAP1A). Low Positive was defined as 1X LoD; medium positive was defined as 3X LoD.

Positive and Negative Agreement: A panel of 150 de-identified clinical specimens was evaluated using the Lab developed C. difficile Direct assay. Lab developed test results were compared to Cepheid Xpert C. difficile and enriched culture results.

Cross-Reactivity: The cross-reactivity panel of 126 different organisms consisted of industry equivalent 106 CFU/mL of bacteria or 105 TCID50/mL of virus in negative stool matrix.

Inhibition/interference: The interference panel was contrived with the ATCC 43255 or NAP1A strain at 4-fold the LoD concentration. Each substance was spiked into the C. difficile contrived stool samples and tested using Lab developed C. difficile Direct.

## RESULTS

Limit of Detection: C. difficile ATCC 43255 LoD was 0.5 CFU/mL. C. difficile NAP1A strain LoD was 1.6 CFU/mL in stool matrix (Table 1).

**Table 1:** *C. difficile* Limit of Detection

Bacterial Strain	(LoD) Concentration	Detection Rate	Average Ct	Maximum Ct	Minimum Ct

ATCC 43255	0.5 CFU/mL	95% (19/20)	39.1	41	38.5
NAP1A	1.3 CFU/mL	100% (20/20)	39.1	42.0	38.4

C. difficile Reproducibility: For ATCC 43255 and NAP1A medium- and low-contrived panels, C. difficile strains were detected in 100% of replicates. Standard deviations were <0.99. Percent coefficients of variation were <3.3 (Table 2).

**Table 2:** Lab developed C. difficile Direct Quantitative Reproducibility

				Between Instrument		Between Operator		Between Run		Within Run		Total	
Channel/Detector	Sample Name	N	Mean Ct	SD	%CV	SD	%CV	SD	%CV	SD	%CV	SD	%CV

C. diff (FAM)	Low Pos 43255	36	38.7	0	0	0	0	0	0	0.87	2.6	0.94	2.7
	Low Pos NAP1A	36	38.8	0	0	0	0	0.7	1	0.76	1.8	0.74	1.9
	Med Pos 43255	36	38	0.31	0.9	0	0	0	0	0.53	2	0.83	2.3
	Med Pos NAP1A	36	37.2	0.16	0.2	0	0	0	0	0.58	1.7	0.52	1.2
	Pos Control	36	31.2	0.27	0.5	0	0	0.1	0.3	0.37	1.6	0.38	1.6
IC (Q670)	Low Pos 43255	36	29.9	0	0	0	0	0	0	0.87	3.1	0.99	3.1
	Low Pos NAP1A	36	29.8	0.28	0.9	0	0	0	0	0.74	2.3	0.76	2.8
	Med Pos 43255	36	29.6	0.26	0.5	0	0	0	0	0.62	2.3	0.69	2.4
	Med Pos NAP1A	36	29.9	0.35	1.5	0	0	0	0	0.6	2.4	0.75	2.6
	Negative	36	30.1	0.12	0.4	0	0	0.22	0.7	0.79	2.8	0.81	2.5
	Pos Control	36	30.8	0.33	1	0	0	0	0	0.7	2.2	0.76	2.9

C. difficile Positive and Negative Agreement: Results from Lab developed C. difficile Direct and Cepheid Xpert C. difficile were in agreement for 95% of positive specimens and 97% of negative specimens (Tables 3 and 4).

**Table 3:** Lab developed C. difficile Direct Positive and Negative Agreement

	Lab developed *C. difficile* Direct vs Cepheid Xpert *C. difficile* (n=150)	Lab developed *C. difficile* Direct vs Enriched Culture (n=110)

**Positive Agreement (Sensitivity)**	95%	93%
**Negative Agreement (Specificity)**	97%	94%

**Table 4:** Lab developed C. difficile Direct Agreement with Cepheid Xpert C. difficile

		Cepheid Xpert *C. difficile*		Total
		Positive	Negative	

**Lab developed *C. difficile* Direct**	**Positive**	32	3	35
	**Negative**	2	113	115
	**Total**	34	116	150

Results from Lab developed C. difficile Direct and enriched culture were in agreement for 93% of positive specimens and 94% of negative specimens (Tables 3 and 5).

**Table 5:** Lab developed C. difficile Direct Agreement with Enriched Toxigenic Culture

		*C. difficile* Enriched Toxigenic Culture		Total
		Positive	Negative	

**Lab developed *C. difficile* Direct**	**Positive**	41	6	47
	**Negative**	3	100	103
	**Total**	44	106	150

Cross-Reactivity: No cross-reactivity was detected with the 126 pathogens tested (subset of representative strains listed in Table 6).

**Table 6:** C. difficile Cross-Reactivity Pathogens Tested in Stool Matrix (Representative Strains)

Abiotrophia defectivear	Candida catenulate	Clostridium septicum
Acinetobacter baumannii	Clostridium bifermentans	Ciostridium tetani
Acinetobacter Iwoffii	Clostridium bolteae	Clostridium difficile (non-toxigenic ATCC43593)
Aeromonas hydrophila	Clostridium butyricum	Desulfovibrio piger
Alcaligenes faecalis subsp. Faecalis	Clostridium chauvoei	Edwardsiella tarda
Bifidobacterium longum	Clostridium fallax	Eggerthellalenta
Campylobacter coli	Clostridium ramosurn	Enterobacter aerogenes
Campylobacter jejuni sub sp .jejuni	Clostridium scindens	Enterobacter cloacae
Candida albicans		

Substance Interference: No interference was detected with the substances tested (Table 7).

**Table 7:** Interferents Tested in Stool Matrix

Substance	Active Ingredient	Starting Interferent Concentration	Final Sample Concentration^a^

Antacid and anti-gas generic	Aluminum hydroxide, magnesium hydroxide	1 mg/mL	0.1 mg/mL
Milk of Magnesia (liquid)	Magnesium hydroxide	2 mg/mL	0.2 mg/mL
Antacid generic	Calcium carbonate	1 mg/mL	0.1 mg/mL
Metronidazole	Metronidazole	140 mg/mL	14 mg/mL
Vancomycin	Vancomycin	14 mg/mL	1.4 mg/mL
Stearic acid	Stearic acid	40 mg/mL	4 mg/mL
Palmitic acid	Palmitic acid	20 mg/mL	2 mg/mL
Barium sulfate	Barium sulfate	50 mg/mL	5 mg/mL
Loperamide hydrochloride generic	Loperamide	0.05 mg/mL	0.005 mg/mL
Pepto-Bismol (liquid)	Bismuth subsalicylate	1.75 mg/mL	0.175 mg/mL
Preparation H	Phenylephrine	20% (w/v)	2% (w/v)
Trojan with nonoxynol-9	Nonoxynol-9	14 mg/mL	1.4 mg/mL
1% hydrocortisone cream generic	Hydrocortisone	20% (w/v)	2% (w/v)
Fleet	Mineral oil	20% (w/v)	2% (v/v)
Laxative generic	Sennosides	1 mg/mL	0.1 mg/mL
Moist towelettes generic	Benzalkonium chloride	100% (v/v)	10% (v/v)
KY Jelly	Glycerin	20% (w/v)	2% (w/v)
Nystatin (6,000 USP units/mg)	Nystatin	100,000 USP/mL	10,000 USP units/mL
Naproxen sodium generic	Naproxen	140 mg/mL	14 mg/mL
Mucin	Mucin	30 mg/mL	3 mg/mL
Whole blood (donor: RH)	Whole blood	50% (v/v)	5% (v/v)
TE (spiked baseline)	None	N/A	N/A

a For each substance, 100 μL of interferent at starting interferent con-centration was added to 900 μL of stool matrix.

## CONCLUSION

Lab developed C. difficile Direct can provide an option for simplified C. difficile testing on the real time PCR. This test was also comparable to both Xpert C. difficile and enriched toxigenic culture for identifying C. difficile.
